# Modulation of CYP1A1 and CYP1A2 Hepatic Enzymes after Oral Administration of *Chios Mastic Gum* to Male Wistar Rats

**DOI:** 10.1371/journal.pone.0100190

**Published:** 2014-06-20

**Authors:** Efrosini S. Katsanou, Katerina Kyriakopoulou, Christina Emmanouil, Nikolas Fokialakis, Alexios-Leandros Skaltsounis, Kyriaki Machera

**Affiliations:** 1 Laboratory of Toxicological Control of Pesticides, Department of Pesticides Control and Phytopharmacy, Benaki Phytopathological Institute, Kifissia, Athens, Greece; 2 Department of Pharmacognosy and Natural Products Chemistry, Faculty of Pharmacy, University of Athens, Athens, Greece; University of Crete, Greece

## Abstract

*Chios mastic gum* (CMG), a resin derived from *Pistacia lentiscus* var. *chia*, is known since ancient times for its pharmacological activities. CYP1A1 and CYP1A2 enzymes are among the most involved in the biotransformation of chemicals and the metabolic activation of pro-carcinogens. Previous studies referring to the modulation of these enzymes by CMG have revealed findings of unclear biological and toxicological significance. For this purpose, the modulation of CYP1A1 and CYP1A2 enzymes in the liver of male Wistar rats following oral administration of CMG extract (CMGE), at the levels of mRNA and CYP1A1 enzyme activity, was compared to respective enzyme modulation following oral administration of a well-known bioactive natural product, caffeine, as control compound known to involve hepatic enzymes in its metabolism. mRNA levels of *Cyp1a1* and *Cyp1a2* were measured by reverse transcription real-time polymerase chain reaction and their relative quantification was calculated. CYP1A1 enzyme induction was measured through the activity of ethoxyresorufin-*O-*deethylase (EROD). The results indicated that administration of CMGE at the recommended pharmaceutical dose does not induce significant transcriptional modulation of *Cyp1a1/2* and subsequent enzyme activity induction of CYP1A1 while effects of the same order of magnitude were observed in the same test system following the administration of caffeine at the mean daily consumed levels. The outcome of this study further confirms the lack of any toxicological or biological significance of the specific findings on liver following the administration of CMGE.

## Introduction

CMG is a resin derived from *Pistacia lentiscus* (L.) var. *chia* (Duham), *Anacardiaceae*, a plant which is mainly cultivated in southern part of Chios Island in Greece. Although CMG is known to have pharmacological activities since ancient times, only recently studies have been conducted to confirm its beneficial effects. Specifically, it has been shown that in *in vitro* conditions CMG has antibacterial and anti-inflammatory effects especially to *Helicobacter pylori* [1]–[4]. Additionally, studies in humans have shown that the consumption of CMG causes significant relief from the symptoms of peptic ulcer [5] as well as inhibiting the activation of *Helicobacter pylori* neutrophil-activating protein, which is involved in the pathogenic effects of *H. pylori* [6]. CMG also presents antiatheromatic and hypolipidemic activity [7] and it can decrease the number of bacteria in saliva and prevent the formation of dental plaque [8] as well as reduce oral malodor and volatile sulfide compounds levels [9]. Moreover, it has been proved by *in vitro* studies that CMG inhibits cell proliferation of cancer cells derived from several types of human neoplasia including mainly prostate, colon [10]–[13], lung [14], [15] and pancreatic carcinoma [16]. The anticancer properties of mastic gum, mainly attributed to constituents belonging to the chemical class of triterpenoids [17], were mainly ascribed to their capability to inhibit cell proliferation through extrinsic and intrinsic apoptosis signaling pathways [17]. Suppression of NF-*κ*B [12] and JAK-STAT signaling pathways, VEGF inhibition, and GSH depletion appear to be potential targets for mastic gum constituents [17]. On the contrary, recently conducted studies, have shown some adverse effects of CMG. Specifically, Kang *et al* (2007) [18] showed that 13-week dietary administration of mastic gum can increase absolute and relative liver weights in rats, along with changes in hematological parameters. Finally, after CMG consumption Doi *et al* (2009) [19] revealed the enhancement of preneoplastic lesions in rat liver using the medium term carcinogenesis bioassay (Ito-test) and among others it showed induction of CYP1A1 enzyme. The above mentioned data clearly show the importance of investigations of the specific effects of CMG on liver enzymes.

CYP1A1 and CYP1A2 enzymes belong to the P450 superfamily and are among the most involved in the biotransformation of xenobiotics and the metabolic activation of pro-carcinogens [20]. Thus, the modulation of these enzymes is considered to be of particular toxicological significance for the human organism. In the present study we sought to investigate whether CMG modulates transcriptional levels of *Cyp1a1* and *Cyp1a2* in rat liver following oral administration of CMG extract of the bioactive compounds, corresponding to the recommended daily consumption of CMG for pharmaceutical reasons and if the potential modulation is also accompanied by changes in enzyme activity of CYP1A1. For the evaluation of potential modulation of CYP1A1/2 on rat liver for human risk assessment, a very well-known bioactive natural compound, caffeine, was studied for detection of potential comparative effects on liver enzymes.

## Materials and Methods

### Ethics Statement

The Laboratory of Pesticides Toxicology, Department of Pesticides Control and Phytopharmacy, Benaki Phytopathological Institute, Greece has an approved Wistar rat breeding and user laboratory animal establishment since 1996 (Reg. No 2538/26-7-1996, modified Reg. No Κ/4620, Κ/4621) (Registration codes EL25BIO027, EL25BIO026). This breeding and user establishment is subject to the authorized guidance and control of Attica Prefecture Veterinary Services regarding the compliance to both national and European legislation regarding the health and welfare of laboratory animals (Presidential Decree No 160/91 Governmental Gazette A′ 64- Recommendation 2007/526/EC Official Journal of the European Communities L197- Presidential Decree No 184/96 Governmental Gazette A′ 137). The establishment always preserves high standards of animal housing, care and welfare of animals. The present study was carried out in February 2012 in compliance with the Presidential Decree 160/1991, Article 3 (Official Gazette A′ 64, 1991) for animal experimentation. The administration of the test substances was carried out under training and educational activities, for which official authorization was not mandatory according to the aforementioned law. An informal ethical group consisting of the veterinarian of the unit, the Director of the Institute and an accredited member of the staff (FELASA C) was established and approved the study. The ethical group approves the experimental protocol and supervises the whole process for studies that official authorization is not mandatory according to the national law that was in force under the period the experiment was carried out.

### Materials

Commercial CMG was purchased from Mastihashop, the official distribution channel of Chios Mastiha Growers Association which is the exclusive worldwide producer of the resin. For the preparation of the CMG total extract that was used in experiments, 1 kg of mastic gum was diluted in 1 L ethyl acetate and then 3 L of methanol were added. The mixture was let stand for 2 days and then the poly-β-myrcene (corresponding to 25–30% of the resin) was decanted. The supernatant solution was filtered and evaporated under vacuum and stored at −20°C.

SYBR GreenER qPCR SuperMix Universal, SuperScript First-Strand Synthesis System for RT-PCR and TRIZOL Reagent were all supplied by Invitrogen. PCR primers were designed with Primer3 (version 0.4.0) and were ordered by Invitrogen. All the other reagents used were readily available commercial products (Sigma Aldrich Europe).

### Experimental animals

Healthy male Wistar rats from the breeding colony of Benaki Phytopathological Institute, were housed in polypropylene cages under controlled environmental conditions (temperature 22±2^ο^C, relative humidity 55±10%, light:dark circle 12∶12 h) and with *ad libitum* consumption of certified food for experimental animals (4RF25, Mucedola, Italy) and water.

### Selection of doses

Six groups of six-month-old male naïve Wistar rats (3 rats/dose, mean weight: 450 g±20%) were orally administered by gavage with 0 (control group), 30 or 100 mg/kg b.w./day of caffeine in two aliquots per day (10 a.m and 3 p.m.) or with 0, 1428 or 2000 mg/kg b.w./day of CMGE at 10 a.m. for three consecutive days. The animals were randomly allocated in each group. CMGE suspension was prepared in carboxymethyl cellulose 1% and administered by gavage using a suitable intubation cannula at 0.7 ml/100 gr bw.

The high dose of CMGE was chosen because it is well above the commercially recommended pharmaceutical dose of *mastic* that can be received by man which is around 1333 mg/person/day (according to the Information leaflet, Chios Mastiha Capsules http://www.gummastic.gr/library/Capsules_Description_ENG.pdf). The dose of 2000 mg/kg b.w./day of CMGE administered in rats corresponds to 2667–2857 mg/kg b.w./day of CMG. When applying a safety factor of 100 (10 for intraspecies variation ×10 for interspecies variation) the respective CMG dose for humans is calculated to be 26.67–28.57 mg/kg b.w./day i.e. 1600–1714 mg/person/day for a 60 kg person. Respectively, the 1428 mg/kg b.w./day dose of CMGE administered to rats corresponds to 1142–1224 mg/person/day which is in the range of the recommended pharmaceutical dose. The low dose of caffeine corresponds to the mean daily intake of caffeine received by an adult man, known to involve hepatic enzymes in its metabolism but with no biological or toxicological significance. The high dose of caffeine corresponds to an excessively high daily intake and was used as a positive control for the activation of CYP1A1 and CYP1A2. The experimental procedure was repeated twice.

### Animal welfare issues - Organ and tissue processing

Several refinement procedures took place in order to ensure that animal welfare issues were properly addressed during the experimental procedures. The procedure was accomplished by well–trained personnel of the experimental unit who has successfully completed a course on Laboratory Animal Science that meets the FELASA category C requirements. All animals had been previously trained in the procedure for 3 days prior to the experiment.

At the end of the dosing period (24 hrs after last treatment), the animals were humanely killed by placing them in an anesthetic chamber and exposing them to an overdose of CO_2_. The euthanasia chamber was gradually filled with CO_2_ (20% of the chamber/min) in order to allow the animals to lose consciousness before CO_2_ causes any pain or distress to rats. The final CO_2_ concentration in the chamber was 80%. Afterwards, the euthanasia chamber was kept open for a few minutes in order to be filled with O_2_ before placing the next animal inside.

Immediately after death, livers were perfused with PBS through the portal vein and 50–100 mg of liver sections were collected, snap frozen in liquid nitrogen and stored at −80°C until further treatment.

### RNA isolation

Total RNA was isolated from the frozen rat liver sections using TRIzol Reagent (Gibco BRL/Invitrogen) according to the manufacturer's instructions. The RNA was quantitated spectrophotometrically at 260 nm (A_260_). The A_260/280_ ratio >1.8 was considered an acceptable measure of RNA purity. RNA integrity was estimated by visual examination of two distinct rRNA bands (28S and 18S) on denaturing 1.2% agarose gel stained with ethidium bromide. Only RNA samples with clear and sharp 28S band about twice as intense as that of 18S, were used for further experiments.

### cDNA synthesis

For the synthesis of the first-strand of cDNA, 1.5 µg of total RNA were reverse-transcribed to cDNA in a total volume of 20 µl, using SuperScript First-Strand Synthesis System for RT-PCR (Invitrogen) according to the manufacturer's instruction using oligo dTs as primers for the reaction.

### Primer design

PCR primers to published gene sequences (GenBank, accession numbers in Table 1) were designed with the Primer3 (version 0.4.0) software. To prevent amplification of sequences from genomic DNA contamination, primers and/or amplicons were designed to cross the exon/exon boundaries (Table 1). To confirm amplification of the expected size fragment, amplification products were characterized by agarose gel electrophoresis.

**Table 1:** Sequence of primers used in real-time PCR, amplicon sizes and annealing temperatures.

Gene accession no.	Sequences	Amplicon size (bp)	Annealing Tm (°C)
rat*β-actin*, NM_031144	Fw: 5′ AACCCTAAGGCCAACCGTGAAAAG 3′ Rv: 5′ CGACCAGAGGCATACAGGGACAAC 3′	110	56
rat*Cyp1a1*, NM_012540	Fw: 5′ TAACTCTTCCCTGGATGCCTTCAA 3′ Rv: 5′ GTCCCGGATGTGGCCCTTCTCAAA 3′	109	56
rat*Cyp1a2*, NM_012541	Fw: 5′ ACCCTGAGTGAGAAGGTGAT 3′	99	56
	Rv: 5′ GAGGATGGCTAAGAAGAGGA 3′		

### SYBR Green RT-qPCR

CYP1A1 and CYP1A2 cDNA were amplified by real-time PCR in the Mastercycler ep realplex real-time PCR detection system with realplex 1.5 software (Eppendorf), using SYBR green as the detection dye. Each amplification reaction was carried out in a total volume of 25 µl containing 12.5 µl SYBR GreenER qPCR SuperMix Universal (Invitrogen), 0.2 µM of each primer and 0.02 µg cDNA for *Cyp1a2* or 0.2 µg cDNA for *Cyp1a1* and β-actin primers. The reactions were cycled 45 times using the following parameters: 95°C for 30 s and 56°C for 1 min during which the fluorescence data were collected. At the end of the PCR, a melting curve was generated by heating the samples from 50 to 95°C in 0.5°C increments with a dwell time at each temperature of 10 s, to verify the specificity of the products. A non-template control was run with every set of primers and no indication of PCR contamination was observed. Lack of PCR products from the non-reverse transcribed RNA control indicated that contamination by genomic DNA did not serve as amplification template.

### PCR data analysis

Expression levels of the target genes were normalized with respect to the reference gene β-actin. To determine the efficiency of PCR amplification of reference and target genes, dilution series (1/10) of cDNA were prepared. Each dilution was amplified in real-time PCR and the obtained threshold cycle (C_t_) values were used to make a graph C_t_ versus log_10_ of the cDNA sample dilution. The slope of the graph was used to determine the reaction efficiency according to the formula: efficiency  =  [10^(−1/slope)^]-1. In each case, only primers presenting efficiency close to 1 were used.

RNAs from each animal were reverse transcribed at least twice. Each cDNA was analyzed at least in duplicate by real-time PCR. Relative gene expression was then calculated using the ΔΔC_T_ method [21]. Statistical significance of differences was assessed by one-way ANOVA followed by Games-Howell post hoc test, using SPSS Version 16.0 for Windows (SPSS BI Greece A.E.); p≤0.05 was considered statistically significant. The statistical calculation was based on 2^−ΔCt^ values.

### Preparation of liver microsomes

After defrosting, livers were thoroughly homogenized (Ultra Turrax T25 digital, IKA) in sucrose buffer (0.25 M sucrose, 4 mL/g wet liver weight). Cell debris, nuclei and mitochondria were initially removed by centrifugation at 10,000 g for 20 min at room temperature (Sorvall Ultraspeed, USA). The supernatant containing the hepatic microsomes was subjected to ultracentrifugation at 100,000 g for 1 h at 4°C (Sorvall Ultraspeed, USA), the pellet was resuspended in 0.1 M Tris–HCl, 0.15 M KCl, pH 7.4 and centrifuged again at 100,000 g for 1 h at 4°C. The microsomal pellet of the second ultracentrifugation was suspended in 0.5 mL of a phosphate buffer solution (0.1 M phosphate salts, pH 7.4) containing glycerol (20% v/v) and EDTA (1 mM), as described in Kuriyama *et al*, 2003 [22] and assessed immediately.

### Enzyme assays

Microsomal protein content was determined according to the method of Bradford, using Bovine Serum Albumin as a standard and measuring absorbance at 595 nm. The selective activity of CYP1A1 was determined by monitoring the formation of resorufin from ethoxyresorufin [23]. More analytically, the reaction mixture contained 5 µM substrate (ethoxyresorufin), 0.1 m µ phosphate buffer (phosphate salts) and 200 µg protein in a final volume of 2 mL and it was equilibrated at 37°C for 2 min in water bath. The reaction was initiated by the addition of 250 µM NADPH in distilled water and the increasing fluorescence was registered in a spectrofluorimeter (Jasco FP-6200, UK) using excitation and emission wavelengths of 550 and 580 nm respectively with a 5 nm band slit width. The activities (pmol resorufin/min/mg protein) were calculated by comparing the rate of increase in relative fluorescence to the fluorescence of known amounts of resorufin, based on the results of 4–6 individual samples in each treatment group. Differences between treatment groups were assessed by Kruskal-Wallis H Test followed by Mann-Whitney U test after Bonferonni adjustment, using SPSS Version 16.0 for Windows (SPSS BI Greece A.E.).

## Results and Discussion

**Figure 1:**
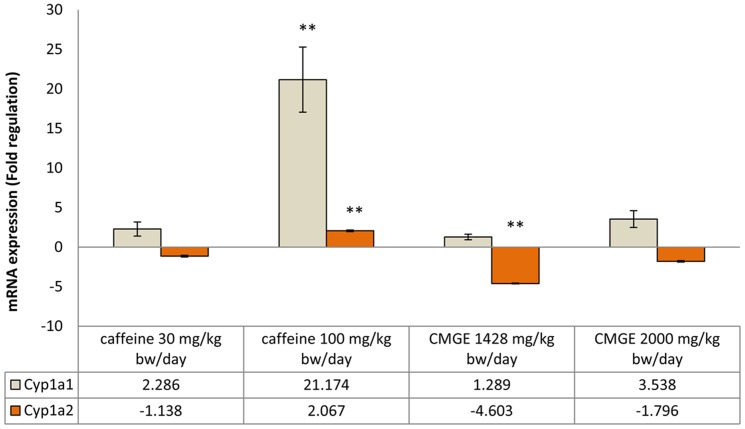
The effect of CMGE and caffeine on the expression levels of *Cyp1a1* and *Cyp1a2* mRNAs in rat liver. The level of mRNA expression (normalized to β-actin) is presented relative to that in animals treated with the vehicle only. Each value represents the mean ± SEM of at least three independent experiments. Statistical significance was assessed by one-way ANOVA followed by Games-Howell post test, **, p<0.01.

The effect of oral administration of the total extract of CMG on the expression of *Cyp1a1* and *Cyp1a2* in rat liver was assessed by using quantitative real-time RT-PCR to measure changes in the mRNA level of these enzymes. Normalization of the measurements was achieved by comparing with the expression of the control gene β-actin, known to be invariant upon treatment with tested compounds. The results of relative quantification are shown in Fig. 1.

CMGE at both doses, failed to induce statistically significant transcriptional change in *Cyp1a1* (p = 0.985 and 0.184, respectively) as compared to caffeine that increased significantly the transcription of *Cyp1a1* when high dose was used (21.174-fold, p = 0.001). It seems that the low dose of caffeine, corresponding to the mean daily intake of caffeine received by an adult man, caused a transcriptional increase (2.286-fold) in the same order of magnitude as CMGE (1.289- and 3.538-fold for low and high dose, respectively). With regard to *Cyp1a2*, medium and high doses of CMGE downregulated *Cyp1a2* transcription (−4.603- and −1.796-fold, respectively) but without a dose response albeit significantly only in the case of medium dose (p = 0.000), whilst the high dose of caffeine upregulated significantly *Cyp1a2* mRNA (2.067-fold, p = 0.000). This result could mean that CMG acts as a “suicide” substrate for CYP1A2 and the consequence of down regulation of this isoform would mean the likelihood of several drug interactions when taken concomitantly with therapeutic drugs that are metabolized primarily by this CYP isoform. Inhibition of *Cyp1a2* could also account for the anti-inflammatory and anticancer activities that have been attributed to CMG. Also, several other isoforms could be involved in metabolism of CMG in the absence of CYP1A1/2. In each case, this downregulation could not be evaluated on its own since it depends also on the balance of the other isoforms and it requires further study.

It is known that gene expression of CYP1A1 is different from that of CYP1A2 since CYP1A1 is primarily expressed in extrahepatic tissues and there is a low expression in the liver [24] whilst CYP1A2 is one of the major isoforms in the rat liver [25]. Indeed, our analysis shows a very low level of *Cyp1a1* in the control liver (Ct >30), confirming that the basal gene expression of CYP1A1 in the liver is low. High dose of caffeine increased mRNA *Cyp1a1* to detectable levels, which could explain why the fold induction is so high.

**Figure 2:**
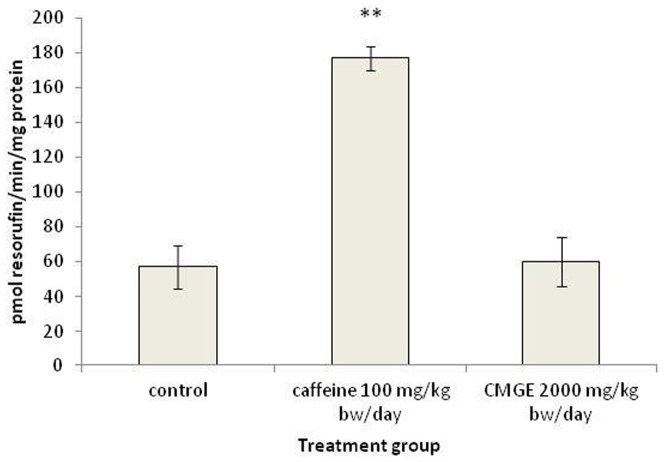
The effect of CMGE and caffeine on the activity levels of CYP1A1 in rat liver. Each value represents the mean ± SEM of at least four samples. Statistical significance was assessed by Kruskal-Wallis H Test followed by Mann-Whitney U test. **:p<0.01

To investigate enzyme activity of CYP1A1, relevant enzyme activities were assessed through the CYP1A1-specific deethylation of ethoxyresorufin [26], as shown in Fig. 2. Microsomes from hepatic tissues of rats exposed to high dose of caffeine (100 mg/kg b.w./day) showed a statistically significant increase in EROD activity in relation to control (3.1-fold). In contrast, high dose of CMGE (2000 mg/kg b.w./day) did not cause a similar induction of activity in relation to control. Caffeine is a quite potent CYP1A1 modulator which has been shown to induce rat hepatic microsomes at high doses [27]. The same dosage scheme to ours caused also a 3.8-fold increase in EROD activity in male Wistar rats [28], which is comparable to the 3.1-fold increase observed in our study. The activity enhancement of CYP1A1 is, almost exclusively, attributed to transcriptional activation through the AhR-dependent signaling pathway [29]. CYP1A1 and 1A2 genes are known to be regulated by the aryl hydrocarbon receptor (AhR) [30]. In intact cells, the unliganded AhR is localized in the cytoplasm, complexed with hsp90 and other proteins. Upon ligand binding, AhR dissociates from its binding partners and enters the nuclei, where it interacts with the AhR nuclear translocator (Arnt) protein. AhR/Arnt heterodimer binds to a specific nucleotide sequence within the enhancer upstream of the CYP1A1/1A2 genes; this process is accompanied by alterations in chromatin structure binding of general transcription factors to promoter and transcription initiation [30]. The only way of CYP1A1 activation known for certain is transcriptional activation through the AhR-dependent signaling pathway [30]. CYP1A2 activation may occur on transcriptional level through AhR-dependent or AhR-independent signal transduction pathways [31], [32] as well as on the post-transcriptional level [33]. Given the promiscuity of AhR ligand binding site, it is not surprising that compounds or extracts of plant-derived materials may induce CYP1A1 activity. Caffeine in particular is supposed to activate AhR and induce AhR-dependent gene expression without competitive binding to it [34]. Caffeine is also known to be a substrate of CYP1A2. On the other hand, the main terpenes of mastic resin limonene, α-pinene, β-pinene [19], have not been proven to be AhR inducers. The former in particular, down-regulated EROD activity in liver microsomes of previously napthoflavone-induced mice [35]. Finally, oleanolic acid, another important constituent of the mastic resin, actually down-regulated CYP1A1 activity in liver microsomes of mice injected with the purified substance [36]. Regarding *Cyp1a2*, α-pinene, β-pinene and limonene did not cause mRNA induction in the liver of Sprague-Dawley rats [37]. Furthermore, oleanolic acid has actually inhibited CYP1A2 in human liver microsomes [38].

Despite the fact that mastic gum is a complex mixture of terpenes and other resin constituents in varied percentages, the results of our study indicate that administration of mastic gum at doses exceeding the pharmaceutical recommended doses did not influence significantly the transcription of *Cyp1a1* and *Cyp1a2*, as well as the enzyme activity of the former. This is an important outcome, given the central role of CYP1A in the oxidative activation of a variety of pro-carcinogens towards reactive intermediates [20]. Further studies are required however to investigate more thoroughly the metabolism of CMG as well as its involvement in alterations of signaling pathways. In a recent study Doi *et al* (2009) [19] reported that DEN-treated F344 male rats had statistically significant increase in mRNA expression of *Cyp1a1* after treatment with mastic resin at a concentration of 1% in diet (corresponding to 500 mg/kg b.w/day). It seems that CMG exhibits a different activity depending on the physiological status of the substrate. Increased *Cyp1a1* mRNA in DEN dosed rats, before dosing with CMG, exaggerated response to CMG.

Summarizing, the results of this first study on naïve animals indicated that administration of CMGE at doses exceeding the recommended pharmaceutical doses did not influence significantly the transcription of CYP1A1 and CYP1A2 enzymes and enzyme activity of the former, which are responsible for the metabolic activation of several pro-carcinogens. The observed modulation of the tested enzymes after CMGE administration is not considered to be of biological or toxicological significance as compared to the respective effects observed after the mean daily human consumption of caffeine. However, further analyses are necessary to investigate more thoroughly the biological effects of CMG as well as its potential beneficial effects.

## Supporting Information

Checklist S1 **ARRIVE Checklist.**
(DOC)
